# Harnessing the Full Potential of Multi-Omic Analyses to Advance the Study and Treatment of Chronic Kidney Disease

**DOI:** 10.3389/fneph.2022.923068

**Published:** 2022-06-27

**Authors:** Claire Hill, Ione Avila-Palencia, Alexander Peter Maxwell, Ruth F. Hunter, Amy Jayne McKnight

**Affiliations:** Centre for Public Health, Queen’s University Belfast, Belfast, United Kingdom

**Keywords:** biomarkers, CKD, data integration, DKD, kidney, multi-omic, review, therapeutics

## Abstract

Chronic kidney disease (CKD) was the 12th leading cause of death globally in 2017 with the prevalence of CKD estimated at ~9%. Early detection and intervention for CKD may improve patient outcomes, but standard testing approaches even in developed countries do not facilitate identification of patients at high risk of developing CKD, nor those progressing to end-stage kidney disease (ESKD). Recent advances in CKD research are moving towards a more personalised approach for CKD. Heritability for CKD ranges from 30% to 75%, yet identified genetic risk factors account for only a small proportion of the inherited contribution to CKD. More in depth analysis of genomic sequencing data in large cohorts is revealing new genetic risk factors for common diagnoses of CKD and providing novel diagnoses for rare forms of CKD. Multi-omic approaches are now being harnessed to improve our understanding of CKD and explain some of the so-called ‘missing heritability’. The most common omic analyses employed for CKD are genomics, epigenomics, transcriptomics, metabolomics, proteomics and phenomics. While each of these omics have been reviewed individually, considering integrated multi-omic analysis offers considerable scope to improve our understanding and treatment of CKD. This narrative review summarises current understanding of multi-omic research alongside recent experimental and analytical approaches, discusses current challenges and future perspectives, and offers new insights for CKD.

## Introduction

Within the last decade, studies have generated a wealth of biological data by exploring the human ‘omes’; from genomics and epigenomics which explore gene variation and modification, transcriptomics which explores gene expression, proteomics and metabolomics which explore the abundance of key biological molecules, to phenomics which explores the potential outcomes or consequences of such biological changes. The valuable insights gained by integrating multiple omic technologies (*via* multi-omics) have improved our fundamental understanding of complex cellular processes, and highlighted how these processes become disrupted during disease. Multi-omic studies have facilitated exploratory analysis of human ‘omes’, improved our basic understanding of their individual function and highlighted important intricate interactions. This knowledge has been harnessed to aid the development of disease biomarkers, the diagnosis of rare disease, the identification of novel drug targets, the design of precision or personalised medicine, and the prediction of disease risk at a population level (1–5).

**Figure 1:**
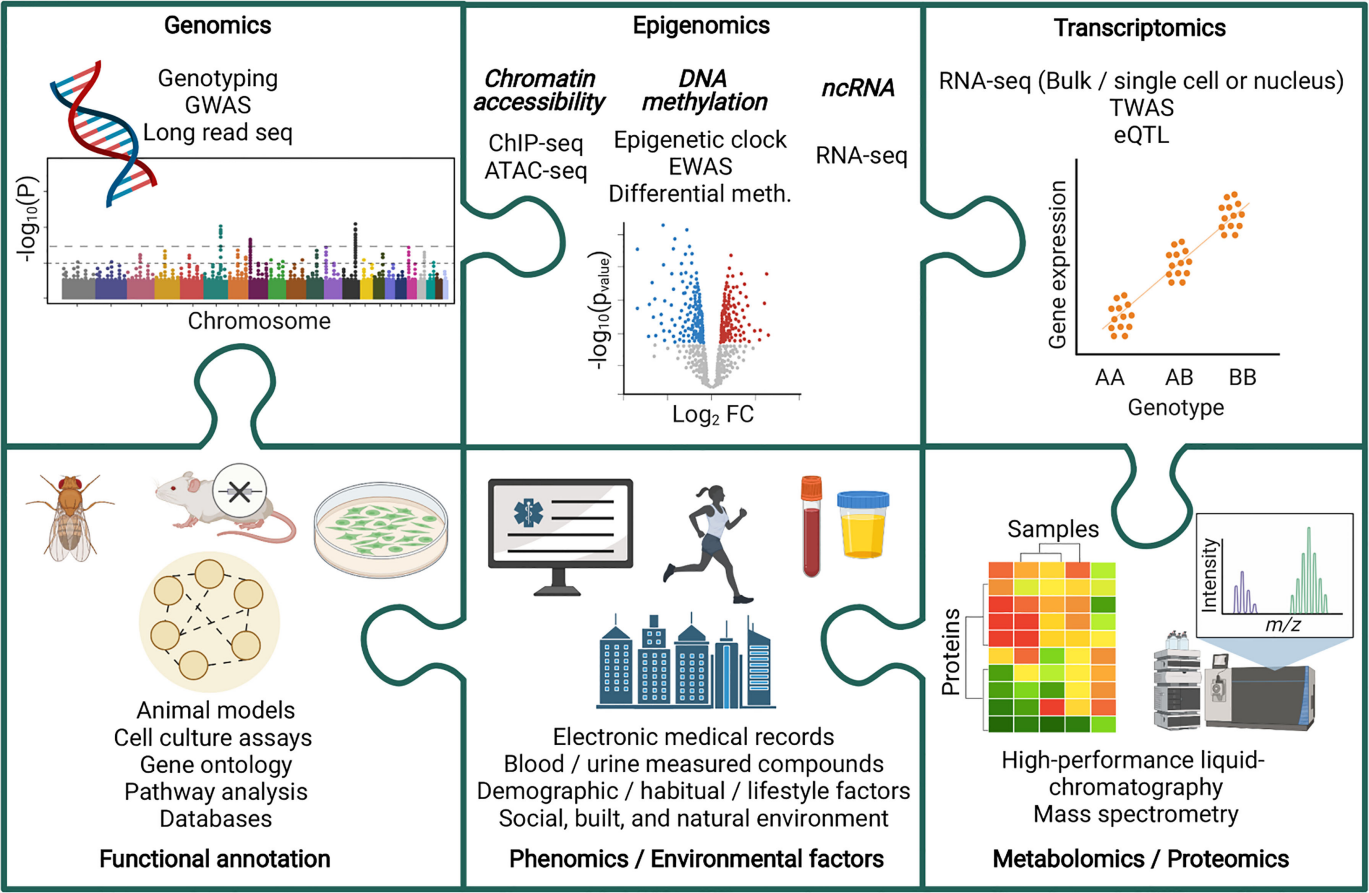
Piecing together the multi-omic puzzle to facilitate the study of chronic kidney disease. ATAC-seq, Assay for Transposase-Accessible Chromatin sequencing; ChIP-seq, Chromatin immunoprecipitation sequencing; eQTL, Expression quantitative trait loci; EWAS, Epigenome wise association study; GWAS, Genome wide association study; Meth, Methylation; ncRNA, non-coding RNA; TWAS, Transcriptome wide association study.

Multi-omic analyses have been harnessed to improve our understanding of chronic kidney disease (CKD) (**Figure 1**). CKD is a non-communicable disease with increasing prevalence worldwide. In 1990, CKD was the 17^th^ leading cause of death, rising to the 12^th^ leading cause of death by 2017, with 697.5 million cases globally that year (6, 7). Further increases in CKD prevalence are expected, with this disease predicted to become the 5^th^ leading cause of death by 2040 (8). In 2017, diabetic kidney disease (DKD) was the leading cause of CKD (7), and in 2018, diabetes accounted for 40% of incident end-stage kidney disease (ESKD) cases in the USA (9). Diabetes is also increasing in incidence worldwide (10, 11), and this, together with increasing prevalence of CKD, is reflective of the aging global population (6). Goal 3 of the United Nations sustainable development goals includes an aim to reduce premature mortality from non-communicable diseases by one third by 2030, with decreasing CKD disease burden highlighted as an important factor in reaching that target (6, 12).

Harnessing a multi-omic approach for the study of CKD is valuable due to the sophisticated contributions from many factors, such as genetic, biological, environmental, lifestyle, social and demographic, to the onset and progression of this disease. Moreover, whilst studying each class of factors individually can provide meaningful insights, proper integration of datasets *via* multi-omic analyses advances our understanding how this network of factors interact to ultimately disrupt biological pathways and influence disease pathology. Advancing our understanding of these complex systems, and how they become disrupted during CKD, has provided opportunities to improve diagnostics, advance treatment strategies, and deepened our fundamental understanding of the causes and effects of CKD, ultimately leading towards our ability to significantly reduce the global impact of this disease.

## Multi-Omics in the Study of Chronic Kidney Disease

The typical five-stage CKD classification system highlights the heterogeneity of this disease, with both genetic and environmental (i.e. encompassing built, natural and social environments factors) influencing CKD onset and progression. Multi-omic analyses have been harnessed to improve our understanding of CKD pathogenesis and progression, as well as how these processes vary between patients, with the ultimate goal of advancing patient care, prioritising resources, and improving patient outcomes.

### Measuring the Influence of Genetic Variation on CKD Pathogenesis and Progression *via* Genomic Analysis

CKD heritability has been estimated between 30% and 75%, depending on the measure of kidney function analysed, such as Glomerular filtration rate (GFR), creatinine clearance or albuminuria; varying also due to the influence of kidney disease risk factors, such as diabetes and hypertension (13–16). Moreover, heritability estimates can vary between studies due to the differences in ethnicity, measurement methods and environment (17). Zhang et al. recently highlighted a knowledge gap in the determination of heritability estimates for CKD (17), identifying that most estimates were determined using familial aggregation in late stage CKD cases only (18–21). These authors presented a large (approximately 150,000 participants of predominantly European ancestry), family-based study of kidney function and carried out the first familial clustering analysis of CKD to include early stages of disease, reporting narrow-sense (additive) heritability estimates ranging from approximately 20% to 50% (17). Interestingly, these authors also reported that those with an affected first-degree relative presented a 3-fold higher risk of CKD compared to the general population, independent of risk factors such as hypertension or diabetes. Those with an affected spouse presented a 1.56-fold higher risk (17). This study highlighted the influence of genetic factors on CKD risk, as well as contributions from environmental influences.

Heritability analyses are useful for guiding the estimates of how much phenotypic variation can be attributed to genetic changes (17). Up to approximately 30% of CKD cases have been attributed to monogenic (single-gene) mutations with strong phenotypic effects; often resulting in early-onset disease (22). More common forms of CKD have been attributed to polygenetic (multiple gene) mutations which cumulatively contribute to kidney function decline; with these patients often presenting with adult-onset disease, subject to variation due to environmental influences (22). Large numbers of genome-wide association studies (GWAS) have been carried out to identify significant associations between specific genetic variants (often single nucleotide polymorphisms, or SNPs) and kidney function or disease (23), recently reviewed by Tin and Köttgen (24). These studies have identified over 250 highly reproducible genetic loci, in both European and non-European populations, associated with GFR (24, 25). Additional GWAS have since been published reporting further genetic variants associated with kidney function (26, 27). For example, Stanzick et al. harnessed a dataset of over 1.2 million individuals to expand the number of genetic loci associated with GFR to 424, with variation within these loci explaining 9.8% variance observed in GFR measurements (26). Zhang et al., however, estimated GFR heritability to be 44% (17), highlighting the phenomenon of missing heritability.

Missing heritability in CKD has recently been discussed by Cañadas-Garre et al. (16) and Anderson et al. (28), with these authors exploring how genomic features such as rare variants, copy number variation (CNV), telomeres, mitochondrial DNA and sex chromosome variation may also contribute to the onset and progression of CKD, accounting for some of this missing heritability. These features have been less extensively studied compared to common autosomal genetic variants. This is largely because the GWAS method is most appropriate for identifying common variants (present in more than 1% to 5% of the population) with moderate effect sizes, which cumulatively contribute towards common phenotypic changes (29, 30). This ‘common disease, common variant’ hypothesis is a fundamental basis of GWAS (29, 31–33). Additionally, rare variants or variants on sex chromosomes have decreased coverage in sequencing arrays commonly used for GWAS analyses, with the added limitation of decreased power when performing sex-specific or rare variant analyses (29, 34). More recently, long read sequencing has provided a promising opportunity to undertake more comprehensive exploration of missing heritability, providing insights into structural and rare variants (35, 36), as well as facilitating adaptive sampling to enrich for sex chromosome analysis (37). Zuk et al., however, propose that due to the influence of genetic interactions, which are often not considered during heritability calculations, the level of missing heritability may in fact be over-estimated (38). This is an important consideration when undertaking genomic analysis, however, this does not invalidate the search for further genetic variants significantly associated with a particular phenotype, but it does highlight the importance of determining the biological function of genetic variants in influencing health and disease, to ultimately improve prevention, diagnosis and treatment (38).

Identifying genetic variants significantly associated with kidney function and disease has also unlocked the potential to explore the causal effects of modifiable risk factors with known genetic associations on these kidney function outcomes. Using Mendelian Randomization methods (39), causal associations between kidney function and factors such as telomere length (40–42), hormone levels (43, 44), coffee consumption (45), macronutrient intake (46), physical activity or sedentary behaviours (47), and education have been identified (48). These studies have great scope to inform about behavioural and environmental changes which may reduce the risk of CKD, or slow disease progression; potentially aiding the discovery of novel ways to moderate the global impact of this disease.

### Epigenetic Modifications Provide an Additional Layer of Variation Influencing CKD Pathogenesis and Progression

Beyond the study of genetic variants which contribute to the onset and progression of diseases such as CKD, the contribution of epigenetic variation has been investigated (23, 49, 50). Epigenetics classically defines changes in gene expression which are not the result of gene mutations but are heritable in the absence of the signal which initiated the change (51). Epigenetics is a broad term commonly used to describe the study of DNA methylation, histone modifications, and non-coding RNA (ncRNA); however, no mechanistic evidence currently exists to confirm the heritability or self-perpetuating capabilities of histone modifications or ncRNA (51–53). DNA methylation of the fifth position of cytosine (5mC) is the most commonly studied epigenetic modification, with these changes having the potential to be both heritable and dynamic in response to stimuli (54–56).

DNA methylation is commonly found within, but not limited to, CpG sites; regions of DNA where a Cytosine residue is followed by a Guanine residue. Of the approximate 29 million CpG sites in the human genome, 60 to 80% are methylated (56). CpG sites are not evenly distributed across the human genome, they accumulate in two types of regions, with 25% of CpGs present within Alu retrotransposons, and 2% within CpG islands (CGIs) (57–59). In vertebrate genomes, over 50% of genes contain CGIs, with other regions of the genome generally CpG-depleted (60). CGIs are present in the promoter region of around 60% of human genes, with these CGIs remaining largely unmethylated (60). CGIs and their methylation status are highly conserved between species (61), for example, 40% of promoter CGIs and 64% of intragenic non-promoter CGIs presented orthologous methylation patterns between mice and humans (62), highlighting the functional importance of these configurations. Indeed, DNA methylation is essential for processes such as X chromosome inactivation (for X chromosome dosage compensation in females) (63, 64), genomic imprinting (65), embryonic development (66), and tissue-specific gene expression (67).

A range of enzymatic factors have been shown to be involved in DNA methylation and demethylation, for example, the DNA methyltransferases, Dnmt3a and Dnmt3b (68). A range of human diseases, such as leukaemia, lymphoma, Tatton-Brown-Rahman syndrome and autosomal dominant cerebellar ataxia have been associated with genetic mutations within the genes encoding these enzymes (66), highlighting the functional importance of DNA methylation. Changes in DNA methylation status have been associated with a wide range of diseases, including cancer, metabolic disorders, autoimmune diseases, and neurological disorders, reviewed by Jin and Liu (69). Environmental factors such as nutritional intake, chemical exposure (pollutants or toxins), and lifestyle can influence epigenetic status (70). Whilst these environmental factors can disrupt epigenetic signals to cause disease, disease states can initiate feedback mechanisms to further alter epigenetic status. For example, when metabolism becomes altered during diabetes (hyperglycaemia) or CKD (uraemia), epigenetic changes can occur which result in altered gene expression, potentially increasing the risk of disease complications; with these processes referred to as metabolic, hyperglycaemic, uremic or inflamed “memory” (71–78).

It is known that advancing age is a risk factor for chronic diseases such as diabetes and CKD (79–86). DNA methylation is an estimator of biological age, as highlighted by the introduction of epigenetic clocks by Steve Horvath and the use of this epigenetic biomarker across multiple tissue and cell types to provide predictions of lifespan and healthspan (87–89). Evidence suggests that accelerated epigenetic aging (an increased difference between chronological and epigenetically predicted age) is associated with CVD, diabetes, Alzheimer’s disease, cancer and kidney disease (87, 90–99). Matías-García et al. performed a trans-ethnic meta-analysis of up to seven populations, investigating five kidney traits (GFR, prevalent CKD, urine albumin-to-creatinine ratio (uACR), microalbuminuria and serum urate) and seven DNA methylation-based age/lifespan predictors (91). These authors identified 23 significant associations between several kidney traits and epigenetic clock age/lifespan predictors; 6 replicated across ethnic groups, and 16 replicated in an ethnic-specific manor (91). Different epigenetic clocks probe different aspects of aging, for example, the extrinsic epigenetic age acceleration (EEAA) clock is a measure of immune system aging (100, 101), whilst mortality risk scores (MRSs) have been associated with oxidative stress (102). CKD is associated with both increased inflammation and oxidative stress (103–108), potentially explaining the strong predictive associations obtained when using these methods in the study of kidney function decline (91).

An alternative method to study epigenetic features of CKD is by harnessing an epigenome-wide association study (EWAS) approach (23, 71). Much like the SNP arrays harnessed for GWAS analysis, commercial arrays have been developed to facilitate the reproducible and high-throughput study of CpG sites across the human genome, for example, the Illumina MethylationEPIC BeadChip Infinium array investigates 853,307 CpG (850K) sites, with increased coverage of regulatory regions compared to previous methylation arrays (109, 110). Alternative forms of epigenetic regulation, such as ncRNA or chromatin modifications, can be analysed *via* methods such as quantitative polymerase chain reaction (qPCR), RNA sequencing (RNA-seq) and chromatin immunoprecipitation sequencing (ChIP-seq) (111, 112), recently reviewed by Walters and Cox (113). Interestingly, computational methods have facilitated the direct detection of epigenetic modifications during Oxford Nanopore genome sequencing (114), identifying a potential avenue for future kidney disease research to intricately integrate and streamline genetic and epigenetic analyses.

Epigenetic variation is associated with CKD and DKD across multiple populations (16, 23, 71, 111, 115–126). Functional annotation of these epigenetic variations has highlighted the potential association of these variations with processes such as haemostasis, endocrine or metabolic control, mitochondrial function, apoptotic cell clearance, immune cell activation, or regulation of cell shape (117, 118, 121, 125, 127). Due to the dynamic and reversible nature of epigenetic medications, such studies may provide attractive targets for therapeutic interventions (128–132).

Functional effects of epigenetic modifications on kidney conditions have been confirmed *via* studies harnessing mouse models, validating effects *in vivo* (133, 134). Park et al. determined that differential methylation of tumor necrosis factor alpha (TNF-α) resulted in altered gene expression, with increased TNF expression in diabetic mice increasing the severity of kidney disease (123). Chen et al. determined that promoter regions of mammalian target of rapamycin (mTOR) regulators were differentially methylated in patients with diabetes (135). These authors highlighted the role of the DNA methyltransferase, DNMT1, in controlling the methylation of mTOR regulator genes, with DNMT1 expression positively correlated with inflammatory activity of peripheral blood mononuclear cells (PBMCs) from diabetic patients (135). Harnessing mouse models, Chen et al. also determined that mTOR dysregulation in diabetic immune cells resulted in kidney inflammation associated with DKD (135).

Specific DNA methylation patterns have also been associated with kidney disease progression and co-morbidities (136, 137), with different methylation profiles observed for early versus late stages of DKD (116), Gluck et al. have shown improved estimations of renal function in DKD patients upon the incorporation of methylation status at CpG sites significantly associated with renal function decline (124). Onishi et al. determined that urine levels of 5-Methyl-2′-deoxycytidine (5MedC), a by-product of DNA methylation, was significantly associated with late-stage CKD prediction (138). Similarly, Marumo et al. determined that *SMTNL2* (Smoothelin Like 2) methylation levels in urine sediment significantly correlated with renal function decline and when incorporated into models to predict faster GFR decline in diabetics, provided a more successful prediction method (120). In a recent study by Dritsoula et al., the relationship between CKD and cardiovascular disease (CVD), a common CKD co-morbidity, was explored in the context of methylation. These authors identified changes in DNA methylation in the arterial wall of CKD patients and uncovered interesting targets for future study to advance our understanding of the molecular dysfunctions occurring in CKD which may result in cardiovascular damage (137). These studies highlight the diagnostic potential of methylation status determination for both disease pathogenesis and progression.

A recent longitudinal study has also explored the effect of various kidney disease treatment methods on methylation levels, in 23 individuals (and 24 controls) with 1 year of follow-up data. Witasp et al. recently identified that the number of significantly differentially methylated CpG sites (compared to healthy controls) fell from approximately 12,000 and 19,000 pre-treatment, to approximately 300 and 400 12 months post-treatment, for dialysis and transplant respectively (139). These authors also noted distinct localisation patterns for differentially methylated CpG sites for dialysis and transplant patients, and highlighted that the methylation status of regions associated with cellular aging or metabolism were particularly altered 12 months post-treatment, to become more in line with healthy control participants (139). 413 differentially methylated genes present in both dialysis and transplant patients remained unaltered 12 months post-treatment, identifying potentially distinct and robust CKD markers warranting future study (139).

### Emerging Insights From Transcriptomic Analysis of CKD

In order to gain additional functional insights into the effects of genetic or epigenetic variants, studies have harnessed transcriptomics, the study of RNA transcripts *via* technologies such as microarrays, qPCR or RNA-seq (140). These investigations have identified gene expression profiles and determined how they differ during health and disease. Many transcriptomic studies investigating kidney disease have focused on examining messenger RNA (mRNA) or the ncRNA subtype, micro RNA (miRNA) (23); however, attention has turned to the contributions made by other ncRNAs such as, ribosomal RNA (rRNA), transfer RNA (tRNA), small nuclear RNA (snRNA), small nucleolar RNA (snoRNA) or long non-coding RNA (lncRNA), with this area of research undergoing recent reviews (141–143). Potential exists to harness RNAs as novel biomarkers for kidney disease pathogenesis or progression, with a distinct focus on urinary RNAs, particularly those contained with extracellular vesicles (protective membrane bound carriers released by cells), as a less invasive and robust means of disease diagnosis (144–148).

A 2018 review summarised literature investigating transcriptomic analysis in the context of kidney disease (23), with a number of additional investigations published in recent years (146, 148–159). An interesting study by Fan et al. carried out RNA-seq analysis of kidney biopsies from early DKD, advanced DKD, or control patients, to reveal gene expression changes from healthy to disease states (158). Gene ontology analysis highlighted that genes involved in iron transport and cell differentiation were positively associated with GFR, whilst genes involved in fibrosis and immune response were negatively associated with GFR (158). Moreover, harnessing kidney single-cell RNA-seq datasets (160, 161), Fan *et al.* deconvolved their dataset to estimate the relative fraction of different kidney cell types within their samples, reporting a significant increase in macrophages, monocytes, fibroblasts, and myofibroblasts in advanced DKD stages, along with a reduction in proximal tubular endothelial cells (158). This transcriptomic analysis reflected results obtained from studies harnessing alternative methods, such as histological examination, which reported increased inflammation and fibrosis during DKD, alongside tubular cell injury (162). Indeed, harnessing single cell RNA-seq has rapidly advanced the field of kidney disease research, recently reviewed by Jiang et al. (163). A greater understanding of how cell heterogeneity changes during kidney disease advances our ability to identify cellular pathways of disease, develop advanced or personalised therapies and improve disease diagnosis or classification (151, 160, 164–166).

### Harnessing Metabolomic and Proteomic Analyses to Aid CKD Diagnosis and Treatment Planning

Alternative methods to study the molecular pathways disrupted during kidney disease involve the investigation of metabolomic and proteomic profiles, recently reviewed by Cañadas-Garre et al. (167). Whilst proteomics assesses the enzymatic, structural protein, antibody, hormonal, DNA-associated or receptor protein profiles, metabolomics assesses the sugar, amino acid, lipid, organic compound or nucleotide profile, which can be impacted by diet or the microbiome (168). These profiles are dynamic and can provide insights into functional changes which occur during kidney disease over time, with genetic, epigenetic or transcriptomic alterations potentially impacting the downstream protein and metabolite landscape. A recent review by Dubin et al. summarised insights gained from the proteomic and metabolomic study of kidney disease, highlighting that because these methods can be easily applied to human blood or urine samples, there is now considerable scope to develop novel biomarkers for disease detection or treatment planning using these approaches (168). Dubin et al. do, however, highlight the challenge of interpreting metabolomic and proteomic studies, with the direction of influence difficult to ascertain (i.e. does the disease cause the protein/metabolite level to change, or vice versa), and the need for downstream functional studies to confirm causative associations (168).

Metabolomic and proteomic studies have resulted in advanced tools to aid patient classification into CKD or DKD stages. Chen et al. utilised ultra-performance liquid chromatography-tandem mass spectrometry to identify five metabolites which explained 94.1% of variation observed between CKD stages (169). Further animal model and cell culture investigations into 5-methoxytryptophan (5-MTP), which presented increased levels in serum as CKD progressed, determined that this metabolite presented strong anti-fibrotic and anti-inflammatory effects, and targeting its regulatory enzyme tryptophan hydroxylase-1 (TPH-1) might prove an effective therapeutic strategy to mitigate CKD progression (169). Fan et al. utilised mass spectrometry to assess the urine proteomic profile of CKD patients without diabetes, DKD patients and diabetic patients without nephropathy, identifying 509 disease specific differentially excreted proteins, and the related pathways, such as late endosomal microautophagy and insulin-like growth factor (IGF) transport regulation in diabetes, and immune system or platelet activation in both DKD and CKD (170). Strong correlations were identified between kidney function measures, such as GFR or uACR, and 46 protein abundance levels, with these authors harnessing urine proteomes to develop models capable of distinguishing between various DKD stages and diabetes (170), with potential future applications within diagnostic testing.

Proteomic or metabolomic studies have been harnessed to assess patient responses to treatment over time, aiding the development of effective and appropriate treatment regimens, unique to each patient. Zhu et al. compared the metabolomic profile of pre-dialysis, haemodialysis and peritoneal dialysis patients, identifying 42 metabolites significantly altered among these three groups (171). Pathway and functional annotation highlighted that haemodialysis and peritoneal dialysis patients had potentially increased risk of infection, increased cardiovascular risk and increased oxidative stress (171). Additionally, Hu et al. carried out metabolomic analysis in haemodialysis patients who had a cardiac death within 1 year of study enrolment, compared to haemodialysis patients surviving after 1 year, with these authors identifying that greater odds for cardiac death were associated with higher levels of several lipid metabolites, an amino acid metabolite and phosphate (172). As highlighted previously, urinary extracellular vesicles have proved useful for the identification of excreted RNAs significantly associated with kidney disease. Proteomic analysis of urinary extracellular vesicles, carried out by Braun et al., identified phosphoenolpyruvate carboxykinase 2 (PCK2) as an early predictive marker of transplant outcome after 1 year (173).

These studies highlight how metabolomic and proteomic studies have uncovered biomarkers to advance the assessment of risk factors and advance the development of non-invasive clinical tests for CKD. Moreover, these studies have advanced CKD treatment planning to aid personalised medicine and improve patient outcomes. With a greater understanding of the functional changes which occur during CKD, metabolomic and proteomic studies have uncovered novel targets to guide future therapeutic development.

### Insights Gained From Environmental Datasets in the Study of Kidney Function and Disease

Beyond the blood, urine or biopsy-derived omic changes associated with kidney disease, researchers have also explored the impact of external factors on CKD. Studies have determined the influence of natural or built environments, pollution and social disparities on CKD. Poverty can impact the development of CKD by modifying health behaviour (due to limited information regarding disease prevention or management), reducing access to healthcare, impacting nutritional intake and increasing exposure to risk factors such as stress, infectious diseases and pollutants (174–177). Closer proximity to open or green spaces has been associated with higher kidney function (178, 179), with the distance to green space shown to increase with social deprivation (180). Closer proximity to open and green spaces may result in improved air quality, increased access to physical recreation and relaxation areas, or reduced noise pollution, with these factors each individually associated with improved kidney function (178, 181–184). Many of these factors change as a result of urbanisation, with urbanisation also shown to modify the presence of potentially toxic elements (PTEs) within the soil, with such PTEs associated with CKD incidence, including CKD attributed to unknown aetiology (185, 186). Urbanisation also modifies the association between air pollutants (namely fine particulate matter and nitrogen dioxide) and CKD (187). Interestingly, the strongest associations between air pollutants and CKD were found in medium-urbanised areas, likely because urbanisation not only results in negative consequences, but also brings positive effects, such as improved healthcare access or higher socio-economic status (187). Scope exists to harness the “PROGRESS” framework (place of residence, race/ethnicity/culture/language, occupation, gender/sex, religion, education, socioeconomic status, and social capital) to study complex environmental, social and demographics interactions, to highlight differences in CKD burden in disadvantaged populations and to identify potential interventions to reach health equity (188).

A number of studies have harnessed a systematic, population level approach to studying environmental impacts on CKD, *via* environment-wide association studies (EnvWAS). Lee at al. assessed bio-monitored chemicals (262 chemicals, measured in blood or urine samples) in participants from the United States National Health and Nutrition Examination Survey (NHANES, 46,748 participants), and identified significant associations with multiple kidney function outcomes; 24 (9%) chemical levels were associated with reduced eGFR, five (2%) with albuminuria, and nine (3%) with composite CKD outcomes (albuminuria or reduced eGFR) (189). These authors determined that increased blood lead and cadmium levels were significantly associated with reduced kidney function, in line with previous studies (189, 190). Interestingly, Yimthiang et al. identified a significant association between simultaneous exposure to cadmium and lead and increased risk of high fasting plasma glucose and kidney function decline, also highlighting the impact of this exposure on DKD progression (191). Cadmium and lead exposure has been associated with processes such as oxidative stress, inflammation and fibrosis within the kidneys (192), with a recent histological analysis by Barregard et al. determining that even low levels of cadmium in the kidney can induce tubular atrophy (193). Here, histological and experimental analyses have be utilised to highlight the biological plausibility of significant EnvWAS associations.

Zheng et al. recently reviewed the strategy and challenges of EnvWAS, highlighting that whilst EnvWAS can provide insights into factors influencing phenotypic changes, care must be taken when drawing associations *via* statistical inference, with further study required to determine the biological function or molecular mechanism of these associations (194). These authors also highlight aspects, such as biomarker or chemical half-lives, spatial or temporal heterogeneity of the environment, detection or quantifications limits of technical methods, and between-factor associations, which can potentially influence EnvWAS outcomes and must be carefully considered during each analysis (194). These studies highlight how a range of data sources can be used to deepen our understanding of the causes and effects of kidney disease, improving our ability to target molecular pathways in novel therapies and advise on the importance of minimising environmental or occupational exposure to specific chemical agents to lower disease risk. This is especially important due to the impact of environmental variation on epigenetic status, with the subsequent cellular effects of these changes potentially resulting in disease or impacting future generations *via* transgenerational epigenetic inheritance (195).

## Challenges Which Remain in the Multi-Omic Study of CKD

Each of the omic analyses discussed above bring their own challenges, such as the effect of confounders or co-variables, the requirement for adequate sample sizes to draw meaningful associations, analytical considerations (significance thresholds, false discovery rates and handling large multi-dimensional datasets). Effective harmonisation and standardised quality control are particular issues for multi-centre studies or those using historical datasets, as well as variations between platform technologies and batch effects. Variability also exists in terms of CKD outcomes, patient characteristics, disease progression and response to treatment, which makes the development of accurate predictive models for CKD prognosis and prediction a challenge (196, 197). Provenzano et al. reviewed the impact of such variability on the study of CKD and highlighted a number of statistical methods and adapted clinical trial designs which can be harnessed to advance the development and assessment of predictive CKD models, and build a more individualised focus to CKD treatment (196, 197). These authors highlight the importance of selecting an appropriate population for model design, which is transferable for use in CKD patients. Moreover, these authors highlight the need for more longitudinal studies with larger datasets to ensure long-term outcomes which may take years to present, such as ESKD or mortality, can be properly assessed (196, 197). Overall, rigorous multi-omic methodologies will advance our ability identify disrupted biological pathways, stratify patients based on risk, prioritise resources, and deliver a personalised treatment approach, ultimately improving patient care and outcomes.

An important consideration for the study of multi-omics is the presence of ‘dark matter,’ consisting of those features which go undetected *via* current methodologies, or those which remain unannotated due to limited prior knowledge (198). For example, coding regions make up only part of human genome (199), with studies now turning to non-coding regions to gain a deeper understanding of the impact of genetic, epigenetic or transcriptomic changes on health and disease (200). Additionally, the Human Metabolite Database (version 5.0) describes 253,243 metabolites; however, as of March 2022, only 24,309 (9.6%) have been detected (with or without quantification) experimentally (201), highlighting a deficit with current experimental metabolomic methods. Interestingly, Odenkirk et al. recently reviewed the application of artificial intelligence methods to advance the annotation of unknowns and improve the estimation of undetected features to improve statistical analysis and interpretation in multi-omic datasets; however, these authors emphasise the need for sufficient model training and downstream validation to ensure confident conclusions can be drawn (198).

### Functional Annotation of Features Significantly Associated With CKD

Functional annotation of those variants or features identified during upstream omic analysis is an important step in the multi-omic pipeline; translating information gained into knowledge which is useful for biological validation, therapeutic development or real-world applications. This is often achieved *via* gene ontology or pathway analysis, with these processes providing insights into functional commonalities and differences, even across different methodologies and cohorts (202–204). Challenges remain in achieving standardisation of pathway analysis annotations between studies and databases, in the unification of similar ontologies to streamline analyses and in advancing annotation coverage (204). An interesting advancement came with the development of an ontology hierarchy annotating the Human Metabolite Database (201, 205), bringing metabolomic studies more in line with the annotations available for genomic and proteomic studies (202, 206, 207). Moreover, text and database mining methods provide additional opportunities to increase the confidence in annotations, and reduce the burden of manual curation of ontology or genotype-phenotype interaction databases (208, 209).

Molecular quantitative trait loci (MolQTL), such as expression (eQTL), methylation (mQTL), splicing (sQTL) or chromatin assembly (caQTL) are genetic variants associated with specific molecular traits. MolQTL were reviewed by Sullivan et al. in the context of CKD, with these authors highlighting how MolQTL are integrated with GWAS to prioritise target genes (210). The Genotype–Tissue Expression (GTEx) project has generated eQTL datasets for 49 human tissues (as of March 2022, release version 8), including the kidney, allowing tissue-specific gene eQTLs to be identified (211). Xu et al. harnessed the GTEx datasets to determine which transcriptionally active SNPs, previously identified *via* GWAS to be associated with CKD disease-defining traits, were associated with kidney-specific or ubiquitous expression (212). This facilitated the prioritisation of genes for downstream functional validation and Mendelian Randomization analysis, which resulted in the discovery that alternatively spliced *MUC1* mRNA isoform expression was causally related to GFR; with alternative splicing a potential allele specific effect (212). This study is an excellent example of how the integration of multiple methodologies can provide biological insights and refine hypotheses to direct downstream studies, such as animal model or cell culture assays, to explore the molecular mechanisms of disease.

### Challenges Faced During the Full Integration of Multiple Omic Datasets

A significant challenge in the field of multi-omics is the full integration of several omic datasets. A number of CKD studies outlined above have carried out single-level analysis, for example, carrying out a separate GWAS, EWAS or TWAS. For multi-level analysis, integration of these datasets must occur, with data integration taking either the form of integrating the same datatype from multiple studies (horizontal integration), or the study of different ‘omes’ within the same cohort (vertical integration) (213, 214). Vertical integration allows a range of methods to be harnessed, each reflecting different aspects of disrupted biological function during disease. For example, proteomics may provide a better understanding of disrupted protein interactions and binding, whereas metabolomics may provide better insights into dysregulated chemical processes. Moreover, ‘ome’ interactions, such as allele specific methylation (215), allele specific chromatin assembly or gene expression (216, 217), and non-coding RNA modulation of protein or gene expression (218), may be overlooked unless a fully integrated multi-omic approach is utilised, meaning vital insights into the molecular mechanisms of kidney disease may go undiscovered.

**Figure 2:**
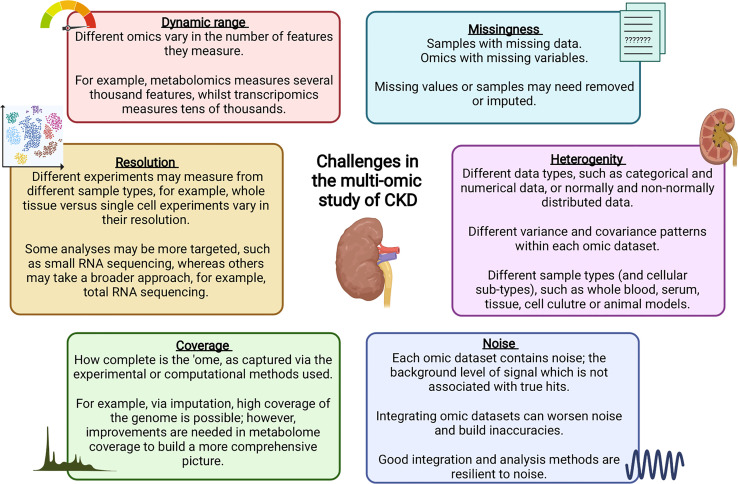
Challenges faced during data integration in multi-omic studies (219–223).

Data integration can take multiple forms, for example, individual omics can be processed separately and integrated later in the pipeline, or alternatively, omic datasets can be concatenated into a single matrix to be processed and analysed together (219). Merging multiple complex and highly variable biological datasets brings with it many challenges, summarised in **Figure 2**; with many tools now available to optimise this process (220). Researchers must ensure the most appropriate and optimal approach is taken, depending on the biological question and the omic datasets available. **Table 1** highlights a number of studies which have utilised an integrated multi-omic approach to study kidney disease.

**Table 1:** Examples of kidney disease studies which have harnessed a more integrated multi-omic analysis approach from 2018.

Title	Summary of methodology	Main findings	Reference	
**Multimodal single cell sequencing of human diabetic kidney disease implicates chromatin accessibility and genetic background in disease progression**	Kidney cortex samples; snATAC-seq (6 HC, 7 DKD); snRNA-seq (6 HC, 5 DKD); Transcribed cis-regulatory element (tCRE) detection (2 HC, 2 DKD); Glucocorticoid Receptor binding/foot printing; Transcription factor motif enrichment; Functional annotation; Validation in alternative dataset.	Increased VCAM1+ injured proximal tubule cells in DKD. These cells present pro-inflammatory expression and transcription factor motifs involved in NF-κB signalling. Allele-specific chromatin changes associated with GFR. Differentially accessible regions enriched for glucocorticoid receptor motifs. Altered chromatin accessibility potentially alters cellular responses during DKD.	(217)	Wilson et al., 2022 (pre-print)
**Assessment of differentially methylated loci in individuals with end-stage kidney disease attributed to diabetic kidney disease: an exploratory study**	Blood derived DNA from up to 253 T1D controls and up to 107 T1D-ESKD patients (depending on comparative analysis); Differentially methylated loci; eQTL and overlapping SNP analysis; Transcription factor motif enrichment; Functional annotation.	Identified associations between differential methylation and T1D-ESKD. Eight top-ranked genes showed eQTL support in a T2D cohort. 13 genes were supported by gene expression and/or methylation data from kidney tubule or glomerular tissues. Top-ranked enrichment pathways included cancer, TGF-β signalling and Th17 cell differentiation.	(118)	Smyth et al., 2021
**Serum integrative omics reveals the landscape of human diabetic kidney disease**	Discovery cohort (n = 1102) containing HC, T2D, Early DKD, and Advanced DKD patients; Proteomics on random 30 samples per group; Metabolomics on complete discovery cohort; ML on metabolomics data to predict DKD status; Proteomics and metabolomics integration to enhance prediction power; Functional annotation; Internal and external validation.	α2-macroglobulin, cathepsin D, and CD324 are protein DKD progression biomarkers. Galactose and glycerolipid metabolism majorly disturbed in DKD, with glycerol-3-galactoside useful in predicting DKD pathogenesis. Integrating proteomic and metabolomic data improved DKD prediction models.	(224)	Liu et al., 2021
**Transcriptome-wide association analysis identifies DACH1 as a kidney disease risk gene that contributes to fibrosis**	TWAS to prioritise genes from two previous eGFR GWAS (n = 765,348 and 280,722); Previous human kidney eQTL data (n = 121); Human kidney RNA-seq (n = 20); Mendelian randomisation; Previous human (n = 10) and mouse (2 P0, 2 adult) snATAC-seq; Previous mouse kidney scRNA-seq (7 healthy); Previous gene expression of microdissected human kidney tubules (n = 95, healthy or disease); ChIP-qPCR (n = 3); Immunostaining; Functional annotation; External validation.	Dachshund homolog 1 (*DACH1*), a cell-fate determination factor, was identified as a kidney disease risk gene.	(152)	Doke et al., 2021
**Mapping the genetic architecture of human traits to cell types in the kidney identifies mechanisms of disease and potential treatments**	Human kidney genotype and RNA-seq (303 glomeruli, 359 tubule); eQTL analysis; scRNA-seq deconvolution; snATAC-seq (n = 2); LD score regression; Previously published GWAS summary statistics for multiple kidney traits; Transcription factor foot printing and motif analysis; Functional annotation; Multi-trait Bayesian colocalization; Drug Gene Interaction Database search; External validation.	Kidney cell-type-eQTLs prioritised proximal tubules for kidney function, and endothelial cells or distal tubule segments for blood pressure. 200 genes were nominated contributors towards kidney function and hypertension association. rs4292 was nominated as a potential variant causing disrupted ACE expression which may result in hypertension and CKD progression.	(165)	Sheng et al., 2021
**A single genetic locus controls both expression of DPEP1/CHMP1A and kidney disease development *via* ferroptosis**	CKDGen eGFR GWAS summary statistics; Human kidney mQTL analysis (n = 188); Previous eQTL in the tubule (n= 121) and glomerular (n = 119) compartments; Previous snATAC-seq (mouse (n =3) and human (n = 2)); Human kidney RNA-seq (n = 432) or Western Blot; Functional annotation; External validation.	Two kidney disease genes, Dipeptidase 1 (*DPEP1*) and Charged Multivesicular Body Protein 1 A (*CHMP1A*), identified as important regulators of ferroptosis, leading to kidney disease development by altering cellular iron trafficking,	(225)	Guan et al., 2021
**Single cell regulatory landscape of the mouse kidney highlights cellular differentiation programs and disease targets**	Mouse kidney snATAC-seq (2 P0, 2 adult), scRNA-seq (1 P0, 1 adult) and whole kidney bulk ATAC-seq (2 P0, 2 3-week old, 2 8-week old); Human kidney snATAC-seq (n = 10); Previous ChIP-seq data; Previous kidney function GWAS; Functional annotation; Motif enrichment; Immunofluorescence.	Chromatin and gene expression changes occur during kidney cell differentiation. Mapping genetic variants associated with human kidney disease onto the mouse cell-specific chromatin landscape implicated specific cell types, developmental stages, genes, and regulatory mechanisms.	(226)	Miao et al., 2021
**Single cell transcriptional and chromatin accessibility profiling redefine cellular heterogeneity in the adult human kidney**	snRNA-seq and snATAC-seq of healthy adult kidneys (n =5); Genotyping and variant annotation of snATAC libraries; Deconvolution of previous bulk RNA-seq (human and mouse); ChIP-qPCR (n = 3); Functional annotation; Immunohistochemistry/Immunofluorescence; External validation.	The activation of NF-κB promotes VCAM1 expression to drive proximal tubule epithelial cell transition (and associated gene expression changes), with the proportion of transitioned cells increasing during kidney injury or disease.	(164)	Muto *at al*., 2021
**Systematic integrated analysis of genetic and epigenetic variation in diabetic kidney disease**	EWAS (CpG methylation) for DKD (250 fast progressing, 250 slow progressing patients); Cell-type expression; mQTL analysis (n = 473); Multi-trait Bayesian colocalization; Mendelian randomization; External validation; Functional annotation.	Forty loci likely mediating kidney function decline associated with inflammation, apoptotic cell clearance and complement activation.	(127)	Sheng et al., 2020
**Integration of GWAS summary statistics and gene expression reveals target cell types underlying kidney function traits**	GWAS summary statistics for four kidney functions (CKDGen Consortium: eGFR n =567,460, BUN n = 243,031, UACR n = 547,361, Urate n = 288,666) or alternative conditions (UK Biobank cohort: Asthma, 28,628 patients, 423,636 controls/CLOZUK 1 PGC2 cohort: Schizophrenia, 40,675 patients, 64,643 controls); RNA-seq of 53 tissues (GTEx); previous scRNA-seq or bulk RNA-seq datasets (from humans, mice or rats); Functional annotation.	Genes associated with kidney function were enriched the in kidney and liver, in particular in the proximal tubule. Enrichment of genes implicated in monogenic glomerular diseases in podocytes.	(227)	Li et al., 2020
**Functional methylome analysis of human diabetic kidney disease**	Microdissected human kidney tubule samples (5 HC, 5 DKD) for whole-genome bisulfite sequencing (WGBS) and RNA-seq; Validation using previous methylation array data (n = 91); Bulk RNA-seq deconvolution; Previous ChIP-seq; Previous mouse kidney scRNA-seq (7 healthy); Functional annotation; Immunohistochemistry; External validation.	Methylation differences occur within the kidney of DKD patients, particularly in the TNF locus, resulting in TNF gene expression changes. Increased TNF levels contributed to disease progression in mouse models, highlighting the potential contribution of this pathway to kidney disease in those with diabetes.	(123)	Park et al., 2019
**Genome-wide association meta-analyses and fine-mapping elucidate pathways influencing albuminuria**	Trans-ethnic GWAS meta-analysis of UACR (n = 564,257), harnessing 54 studies; PheWAS harnessing electronic medical records (n = 192,868); Previously published RNA-seq datasets for 44 tissues (GTEx), kidney cortex (The Cancer Genome Atlas, n = 99) or human kidneys (n = 96); Previous microarray expression data for microdissected glomerular and tubulointerstitial tissues (n =187); eQTL analysis; pQTL (n = 3301); Previous genome-wide summary statistics for genetic correlation; Functional annotation.	68 UACR-associated loci, with PheWAS revealing associations with proteinuria, hyperlipidaemia, gout, and hypertension. Differential expression levels (RNA or protein) observed for UACR-associated genes in the kidney. Knockdown of prioritised genes (*OAF* and *PRKCI*) in Drosophila nephrocytes reduced albumin endocytosis, highlighting novel pathways potentially important for albuminuria.	(228)	Teumer et al., 2019
**Mapping eGFR loci to the renal transcriptome and phenome in the VA Million Veteran Program**	Meta-analyses of eGFR GWAS (n = 280,722), stratified by ancestry, diabetes status, and hypertension status; Replication in a trans-ethnic GWAS meta-analysis of eGFR (n = 765,289); PheWAS (n = 192,868); Previous human eQTL datasets (microdissected kidney tissues (n = 151), kidney cortex (The Cancer Genome Atlas (n = 99) and 121 tubule/glomerulus samples); Previous scRNA-seq murine kidney (7 healthy) dataset; Genetic risk score.	82 previously unreported variants and confirmed 54 loci associated with eGFR, with consistency observed across ancestries. Genetically predicted gene expression and eGFR association revealed 36 previously unreported and 9 known genes, with gene expression mapping to renal cell types. An eGFR genetic risk score was associated with several kidney disease-related phenotypes.	(229)	Hellwege et al., 2019
**The Use of Targeted Next Generation Sequencing to Explore Candidate Regulators of TGF-β1’s Impact on Kidney Cells**	Mouse primary mesangial cells (TGF-β1 and control treated (n = 3)) underwent miRNA-seq and RNA-seq (from total RNA extractions); MeDIP-Seq (DNA Methylation); ChIP-Seq (H3K27me3 methylation); Functional annotation.	Confirmed the regulation of DNA methylation and H3K27me3 after TGF-β1 treatment of kidney cells in culture. KLF7 and Gja4 expression levels were linked to DNA methylation during TGF-β1 treatment, suggesting that TGF-β1 regulates these two genes. Identified the association between epigenetic changes and expression of genes related to kidney injury.	(230)	Wang et al., 2018

ATAC-seq, Assay for Transposase-Accessible Chromatin sequencing; BUN, Blood urea nitrogen; ChIP, chromatin immunoprecipitation; CKD, Chronic kidney disease; DKD, Diabetic kidney disease; eGFR, Estimated Glomerular Filtration Rate; eQTL, Expression quantitative trait loci; EWAS, Epigenome-wide association study; GTEx, Genotype-Tissue Expression project; GWAS, Genome wide association study; HC, Healthy control; MeDIP-seq, Methylated DNA immunoprecipitation sequencing; ML, Machine Learning; mQTL, Methylation quantitative trait loci; NF-κB, nuclear factor kappa-light-chain-enhancer of activated B cells; PheWAS, Phenome wide association study; pQTL, Protein quantitative trait loci; qpCR, quantitative polymerase chain reaction; RNA-seq, RNA sequencing; sc, single cell; sn, single nucleus; T1D, Type 1 Diabetes; T1D-ESKD, Type 1 Diabetes with End Stage Kidney Disease; T2D, Type 2 Diabetes; TGF, transforming growth factor; TNF, Tumor necrosis factor; TWAS, Transcriptome wide association study; UACR, Urine Albumin-to-Creatinine Ratio.

### Improving Cohort Phenotype Information to Improve Multi-Omic Analyses of CKD

An additional aspect of multi-omic studies which holds potential to advance the analysis and interpretation of data is carefully phenotyped cohorts. For multi-omic studies, particularly those carried out between cohorts or consortia, lack of standardisation in the measures and classification of kidney function or co-variables can limit the Findability, Accessibility, Interoperability, and Reuse (FAIR) of datasets (231). Clinical, epidemiological and demographical records provide a wealth of data, and with careful and proper integration into multi-omic datasets, they can provide advanced phenotypic information, recently reviewed by de Maturana et al. (232). Shang et al. provide an excellent demonstration of harnessing clinical records to study CKD; developing an algorithm capable of classifying patients into CKD stages based on electronic clinical records (233). *Via* an observational study, these authors determined the presence of co-morbidities across CKD stages, identifying novel associations, such as the prevalence of several psychiatric comorbidities among patients with mild CKD compared to those patients with normal renal function, independent of age and sex (233). These authors also utilised their algorithm in the Electronic Medical Records and Genomic (eMERGE) network, carrying out a GWAS analyses of CKD (Stage 3 or greater) which identified significant associations with *UMOD* and *APOL1* (233, 234). An additional Phenome-wide association study (PheWAS) identified further associations for these genes with a number of additional kidney associated phenotypes, such as kidney transplantation, ESKD, and dialysis (233). The methods developed by Shang et al. have the potential to support the provision of personalised medicine for those living with kidney disease; facilitating risk stratification for optimised treatment planning. These authors do highlight the need for further investigations to be conducted in adult and child cohorts, across diverse ancestral backgrounds (233), to ensure that the tools developed are appropriate for use across all patients, in a fair and equitable manner.

### Considering Sex Imbalances in the Multi-Omic Study of Kidney Disease

Whilst future studies must incorporate the assessment of CKD across diverse ancestral backgrounds, work is also required to assess CKD between sexes. In 2017, the age-standardised prevalence of CKD was higher in females (9.5%) compared to males (7.3%), whereas the age-standardised incidence of dialysis and transplantation was higher in males (13.7 per 100 000 population) compared to females (8.6 per 100 000 population) (7). Moreover, the global age-standardised CKD mortality rate was 1.39 times greater in males compared to females (7), suggesting that whilst more females are diagnosed with CKD, males may have faster disease progression. Sex-specific variations in omic datasets are potentially overlooked when both sexes are analysed in a single analysis. Bond et al. highlight that sex biases within curated databases may also result in uninformative or lower significance results in scenarios where sex differences exist (235). These authors provide action points for scientists, databases and funding agencies to tackle this problem, adding that including sex as a covariate in a mixed cohort analysis is insufficient to account for this fundamental variable, with the need for analyses to be run in both mixed and sex-stratified groups to optimise data analysis and interpretation (235).

A small number of sex-stratified analyses of the healthy kidney and kidney disease have been carried out (236–238), however, more work is needed in this area. Graham et al. recently determined that S*LC47A1* (also known as *MATE1* (multidrug toxin and extrusion protein 1)) was significantly associated with GFR in females but not in males (239). This gene presented cell-type specific expression in the mouse kidney proximal tubule, with previous studies also supporting hormonal regulation of this gene (239–241), highlighting biological plausibility that this gene may influence sex-specific features of kidney disease. Sex chromosomes may also contribute to sex-specific pathogenesis and progression of CKD; however, more advanced sex chromosome imputation and wider inclusion of sex chromosome genes on genotyping or methylation arrays is required to gain further insights from these largely ignored chromosomes (16, 28). Mosaic loss of the Y chromosome (mLOY), which increases with age and has been strongly associated with both diabetes and cardiovascular disease (242), two common CKD comorbidities, has been explored in the context of renal cancers (243, 244), but not CKD. This work highlights an interesting focus for future study, which may uncover novel sex-specific mechanisms of CKD pathogenesis, potentially useful for the development of novel diagnostics or personalised therapeutic targets.

## Linking Causes and Consequences of CKD Together

**Figure 3:**
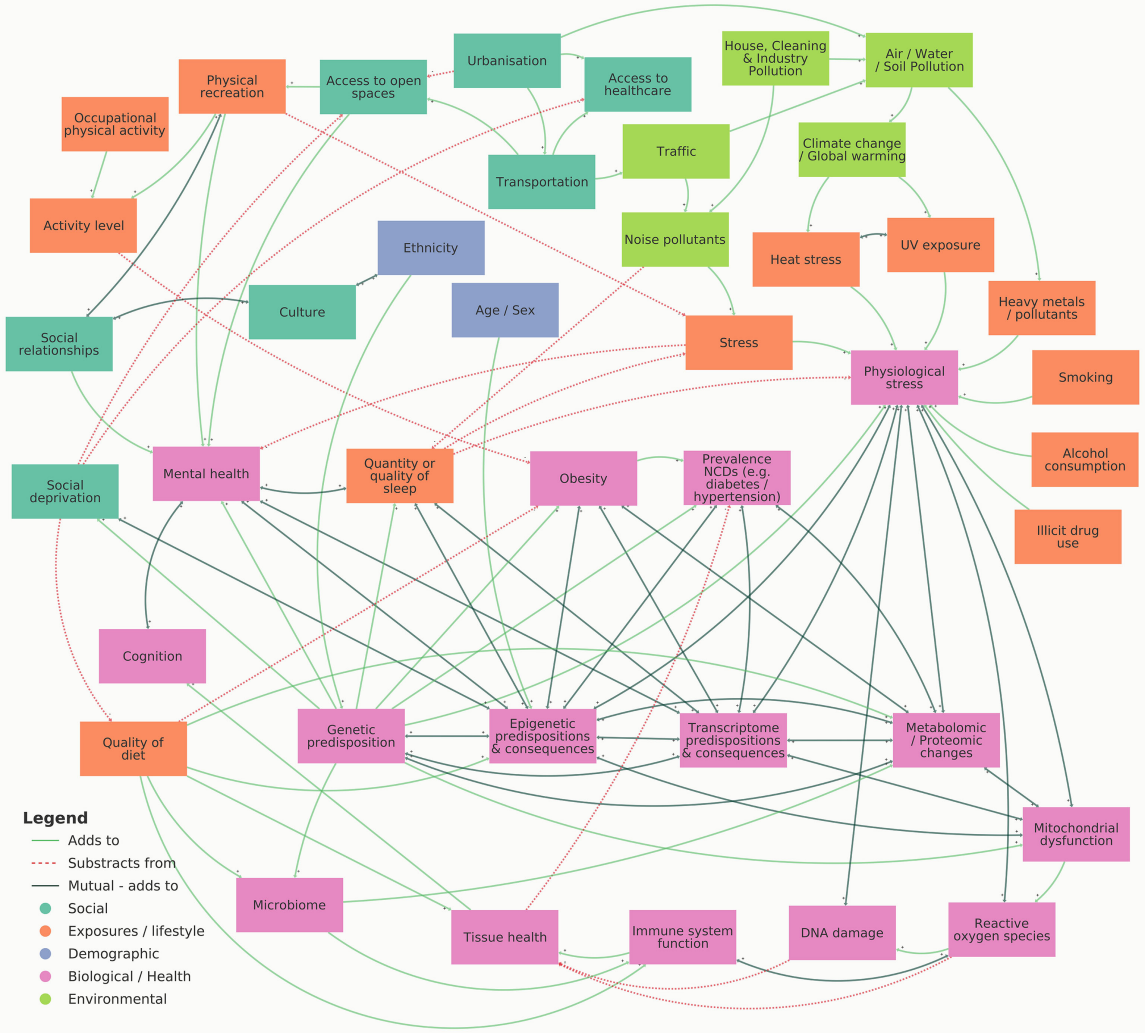
Visual representation illustrating the influences and consequences of multi-omic biomarkers on CKD. An interactive version of this map can be viewed online at https://embed.kumu.io/4ac1a6b9dfef3b8bc1ffdf165737c6c4.

This review highlights multiple forms of omic analyses which have been conducted to investigate the factors influencing CKD, such as genomics, epigenomics, transcriptomics, metabolomics and proteomics, phenomics, as well as exploring social and environmental impacts. The visual representation shown in **Figure 3** summarises influences and consequences of multi-omic biomarkers on CKD and associated variables. Whilst many studies have explored single-level omic analysis, significant value can be achieved by harnessing multi-level analysis by combining numerous omic datasets. Various challenges exist when fully integrating multiple omic datasets for the study of CKD, such as optimising data merging, incorporating the study of sex-biases, and improving nomenclature and phenotyping. These processes must be properly considered for each analysis to enhance data analysis and improve interpretation. Moreover, by understanding these challenges prior to experimental design, more effective data and meta-data generation can be achieved, aiding data sharing and accessibility between studies, ultimately accelerating this research field.

## Conclusion

CKD is a heterogeneous disease presenting a significant impact on global healthcare budgets. Decreasing the burden of CKD has been identified as an important factor in achieving the United Nations sustainable development goal of reducing premature mortality from non-communicable diseases by one third by 2030. In order to achieve this target, a range of approaches have been taken to better understand CKD pathogenesis and progression. Multi-omic studies have facilitated the discovery of novel genetic and epigenetic variants significantly associated with CKD, with gene ontology or pathway analysis, as well as data mining, facilitating the prioritisation of those targets significantly associated with kidney function decline. The functional consequences of such variations have been assessed *via* transcriptomic, proteomic, metabolomic and phenomic analyses, as well as by harnessing *in vitro* and *in vivo* models. Whilst challenges remain in the comprehensive integration of such complex and multifaceted biological datasets, a multi-level approach to combining such datasets has facilitated the discovery of novel pathways associated with CKD pathology, provided insights into the biological effect of different treatment routes, and improved our understanding of disease progression at an individual patient level. Many opportunities remain in the field of multi-omics, with improved machine learning, DNA or RNA sequencing, molecule detection, data analysis and statistical tools being developed, applicable for studying a range of human diseases, including CKD. The multi-omic study of CKD has thus far aided the identification of new therapeutic targets, paved the way towards personalised treatment plans and advanced our knowledge of risk factors for CKD and its progression, with the ultimate goal of improving patient care and outcomes.

## Author Contributions

CH & AMK conceived the review. CH initially drafted the review with input from AMK. AM provided critical clinical oversight while IA-P and RH supported the development of **Figure 3**. All authors contributed to the article, reviewed the manuscript for important intellectual content and approved the final version for submission.

## Funding

This research is supported by the HSC R&D division (STL/5569/19) and UKRI (MRC MC_PC_20026). CH is supported by funding from a Science Foundation Ireland and Department for the Economy, Northern Ireland partnership award (15/IA/3152). IA-P is supported by funding from the UKRI Industrial Strategy Challenge Fund (ESRC, SBDRP, ES/V016075/1).

## Conflict of Interest

The authors declare that the research was conducted in the absence of any commercial or financial relationships that could be construed as a potential conflict of interest.

## Publisher’s Note

All claims expressed in this article are solely those of the authors and do not necessarily represent those of their affiliated organizations, or those of the publisher, the editors and the reviewers. Any product that may be evaluated in this article, or claim that may be made by its manufacturer, is not guaranteed or endorsed by the publisher.
